# DCP-YOLOv7x: improved pest detection method for low-quality cotton image

**DOI:** 10.3389/fpls.2024.1501043

**Published:** 2024-12-19

**Authors:** Yukun Ma, Yajun Wei, Minsheng Ma, Zhilong Ning, Minghui Qiao, Uchechukwu Awada

**Affiliations:** ^1^ School of Software, Henan Institute of Science and Technology, Xinxiang, Henan, China; ^2^ School of Information Engineering, Henan Institute of Science and Technology, Xinxiang, Henan, China; ^3^ Huanghe Jiaotong University, Jiaozuo, Henan, China; ^4^ School of Mechanical and Electrical Engineering, Henan Institute of Science and Technology, Xinxiang, Henan, China

**Keywords:** low-light environments, cotton pest, YOLOv7x, object detection, image enhancement

## Abstract

**Introduction:**

Pests are important factors affecting the growth of cotton, and it is a challenge to accurately detect cotton pests under complex natural conditions, such as low-light environments. This paper proposes a low-light environments cotton pest detection method, DCP-YOLOv7x, based on YOLOv7x, to address the issues of degraded image quality, difficult feature extraction, and low detection precision of cotton pests in low-light environments.

**Methods:**

The DCP-YOLOv7x method first enhances low-quality cotton pest images using FFDNet (Fast and Flexible Denoising Convolutional Neural Network) and the EnlightenGAN low-light image enhancement network. This aims to generate high-quality pest images, reduce redundant noise, and improve target features and texture details in low-light environments. Next, the DAttention (Deformable Attention) mechanism is introduced into the SPPCSPC module of the YOLOv7x network to dynamically adjust the computation area of attention and enhance the feature extraction capability. Meanwhile, the loss function is modified, and NWD (Normalized Wasserstein Distance) is introduced to significantly improve the detection precision and convergence speed of small targets. In addition, the model detection head part is replaced with a DyHead (Dynamic Head) structure, which dynamically fuses the features at different scales by introducing dynamic convolution and multi-head attention mechanism to enhance the model's ability to cope with the problem of target morphology and location variability.

**Results:**

The model was fine-tuned and tested on the Exdark and Dk-CottonInsect datasets. Experimental results show that the detection Precision (P) of DCP-YOLOv7x for cotton pests is 95.9%, and the Mean Average Precision (mAP@0.5) is 95.4% under a low-light environments, showing improvements of 14.4% and 15.6%, respectively, compared to YOLOv7x. Experiments on the Exdark datasets also achieved better detection results, verifying the effectiveness of the DCP-YOLOv7x model in different low-light environments.

**Discussion:**

Fast and accurate detection of cotton pests using DCP-YOLOv7x provides strong theoretical support for improving cotton quality and yield. Additionally, this method can be further integrated into agricultural edge computing devices to enhance its practical application value.

## 1 Introduction

China is one of the world’s largest producers and consumers of cotton (Chen, 2024), which plays a pivotal role in China’s agricultural and textile industries. However, various pests often threaten cotton during its growth process (Li et al., 2024a). Some pests hinder plant growth by feeding on cotton leaves and bolls, while others suck cotton sap and transmit diseases, resulting in disease or death of cotton. On the contrary, cotton-beneficial insects reduce the reproduction and spread of pests by feeding on or parasitizing them, and some of them also feed on weed leaves to control weed growth and promote the development of cotton ecosystems. Therefore, detecting and distinguishing cotton pests and beneficial insects is crucial for production. With the modernization and intelligence of agriculture, using deep learning object detection methods to detect insects quickly and accurately provides a powerful tool for pest control in cotton fields.

Despite the significant progress of deep learning in object detection, in the practical application of cotton pest detection, complex natural conditions, especially low-light environments, can cause some adverse effects on the detection task. In low-light environments, the acquired image noise increases, brightness and contrast decrease, and problems such as blurring of image details and underexposure may occur, leading to difficulties in effectively extracting and recognizing pest features, and the degradation of image quality seriously affects the performance of the detection system. Existing deep learning object detection models, such as the YOLO (You Only Look Once) (Terven et al., 2023) series, perform well under standard lighting conditions. However, their performance in low-light environments is significantly reduced. Therefore, designing or improving the object detection model to overcome the underexposure and noise problems, improve the precision and robustness of pest detection, and ensure the yield and quality of the crop has become an urgent problem.

Currently, some low-light object detection methods use enhancement algorithms to improve image quality. However, these methods often amplify image noise, which negatively impacts the subsequent detection performance. Other studies have modified object detection network architectures to enhance feature extraction capabilities, which, to some extent, improve detection performance in low-light environments. However, these approaches still fail to cope with extremely dark conditions, making it difficult to ensure detection accuracy and reliability.

By combining advanced image enhancement techniques with optimized deep learning object detection algorithms, this study solves the challenges of low image quality, difficulty in pest feature extraction, and low detection accuracy of models in low-light environments for cotton pest detection. we first employ a denoising network to remove noise from low-quality images, and then use low-light image enhancement methods for further unpaired training to improve image quality. This approach effectively mitigates the impact of noise on detection accuracy while enhancing image contrast and brightness, overcoming the limitations of existing low-light object detection methods. For the object detection network, we introduce an attention mechanism, optimize the loss function, and improve the detection head design, further enhancing the model’s feature extraction capabilities. By jointly optimizing both image enhancement and network improvements, this method provides an effective and accurate solution to the challenge of cotton pest detection in low-light environments, even under extreme conditions.

Overall, our main contributions can be summarized as follows:

- A new module, DA-SPPCSPC, is proposed to replace the SPPCSPC (Li et al., 2023) module in the Neck part of the YOLOv7x network architecture. Add the DAttention (Xia et al., 2022) mechanism after the last convolutional layer of the SPPCSPC module, by dynamically adjusting the attention weights, the model’s attention is focused on the weak features, such as the edges and textures of the target insects, reducing the effect of insufficient light and improving the model’s feature extraction ability, which leads to a higher detection precision.
- For the pest detection task, the loss function of the YOLOv7x model is optimized by replacing the original CIOU (Complete Intersection Over Union) (Zheng et al., 2020) loss with the NWD loss (Wang et al., 2021) in the base loss calculation module and replacing the CIOU loss with the NWD loss in the optimal transmission allocation loss calculation module, then combine it with the OTA (Optimal Transport for Label Assignment) (Li et al., 2024b) strategy. The NWD loss improves the regression precision of the bounding box by calculating the normalized Wasserstein distance between the prediction box and the ground truth box in terms of width, height, and center position, which improves the model detection performance and accelerates the convergence speed when dealing with small and dense targets like insects.
- Replacing the detection head of the model with a DyHead (Dai et al., 2021) structure, which can adaptively adjust the feature extraction strategy by combining the dynamic convolution and the three attention mechanisms, effectively enhances the target feature representation in low-light environments and enables the model to distinguish better the color difference between the target and the background, and more accurately detects the target’s variable morphology and location.

## 2 Related work

With the rapid development of computer vision technology, object detection (Hui-lan, 2020), as one of the core tasks in computer vision, has made significant progress and wide application. In recent years, the emergence of deep learning algorithms, especially CNN (Convolutional Neural Network) (Li et al., 2022b), has strongly supported the development of the object detection field.For instance, the work on (Jiang et al., 2018) demonstrates how the incorporation of decoupled attention mechanisms within CNNs can significantly improve feature extraction, particularly in weakly supervised scenarios, by better focusing on relevant regions of the image. Two-stage object detection algorithms, such as R-CNN (Region CNN), Fast R-CNN, and Faster R-CNN (Bharati and Pramanik, 2020), have greatly improved the object detection performance of computer vision by introducing the RPN (Region Proposal Network), which significantly improves the detection precision and speed.On this basis, methods like (Jiang W. et al., 2021), based on Faster R-CNN, enhance detection under weak supervision by using dynamic sampling strategies to refine region proposals. In addition, the proposal of one-stage detection models such as YOLO series and SSD (Single Shot MultiBox Detector), (Liu et al., 2016) further simplifies the detection process and becomes a mainstream method in object detection. Nowadays, more and more research has been conducted to improve and optimize the baseline. For example, by introducing feature pyramid structures such as FPN (Feature Pyramid Networks) (Luo et al., 2022), PANet (Path Aggregation Network) (Piao et al., 2023), and attention mechanisms such as Biformer (Vision Transformer with Bi-Level Routing Attention) (Liang and Yuanjun, 2024), CPCA (Channel Prior Convolutional Attention) (Luo and Tian, 2024) to achieve more precision object detection in complex backgrounds and dynamic environments. The continuously improved object detection algorithms have been widely used in the fields of autonomous driving (Qian et al., 2023), facial recognition (Wang et al., 2024b), medicine (Ragab et al., 2024), etc., which have promoted technological progress and practical applications in related industries.

Although the above methods perform well on high-quality datasets with normal light levels, the performance of the generalized object detection model degrades significantly in low-light environments. The low-light environments significantly reduce the contrast and brightness of the image, making it difficult for the target features to be effectively captured and significantly increasing the model’s false and missed detection rates. In addition, the noise in the low-quality image also blurs the target object edges and texture details, seriously affecting the detection model’s feature extraction capability. Therefore, the limitations of generalized models highlight the necessity of low-light object detection.Similar challenges are encountered in other adverse conditions, as shown in (Ni et al., 2023), which improves detection accuracy under adverse weather conditions by dynamically adjusting feature extraction mechanisms based on the environment.This research highlight the importance of developing adaptive models that can handle varying conditions.

Several studies have been conducted in the field of low-light object detection. (Irtiza et al., 2022) evaluated the performance of several existing deep learning models [YOLOv7 (Wang et al., 2023a), DETR (Zhu et al., 2020), RetinaNet (Lin et al., 2017), and EfficientNet (Tan and Le, 2019)] on customized low-light pest datasets. Although the results showed that YOLOv7 performs best in low-light environments, the detection performance and robustness of these models need to be further enhanced. In recent years, some new research in low-light object detection has focused on improving the network structure of object detection models. A study (Xiao et al., 2020) proposed a night vision detector based on RFB (Receptive Field Block) (Liu et al., 2017) to address issues such as the effectiveness of object detection in low-light environments. It combined a specifically designed feature pyramid network and context fusion network to improve the precision of low-light object detection. (Li et al., 2019) proposed an improved FDN model specifically designed to solve the problem of poor coal mine underground environment with an improved Faster RCNN algorithm for pedestrian detection problems. The method uses a deep convolutional neural network to extract features from images automatically, and by introducing the RPN structure and feature fusion techniques, it is effective in low-light pedestrian detection and handling blur. Although these methods have some effectiveness in low-light environments, if the lighting conditions are highly dim and the exposure is severely insufficient, simply relying on improving the network structure to enhance the feature extraction capability, like the above methods, can only partially solve the problem. In order to further improve the detection performance, it is necessary to improve the quality of the low-quality image to provide a more precise and more accurate input image for the object detection algorithm.

Low-light image enhancement aims to improve the quality of images captured under low-light environments to make them more suitable for human eye observation and subsequent computer vision tasks. Significant progress has been made in the field of low-light image enhancement. Some researchers have combined Retinex theory and deep learning algorithms to propose RetinexNet (Wei et al., 2018), which utilizes an end-to-end network for low-light enhancement and better maintains the natural colors and details of the image. EnlightenGAN (Jiang Y. et al., 2021) enhances the brightness, contrast, and details of low-quality images through adversarial training of the generator and the discriminator. (Tian et al., 2023) provided a comprehensive review of deep learning-based low-light image enhancement algorithms, pointing out that low-light image enhancement not only improves the visual effect of images but also significantly improves the performance of downstream computer vision tasks, including object detection. Therefore, some studies have attempted to combine low-light image enhancement algorithms with object detection models. (Wang et al., 2023b) optimized the structure of the YOLOv5 object detection network by combining low-light enhancement algorithms to improve the performance of object detection within a specific scene of a low-light mine. IDOD-YOLOV7 (Qiu et al., 2023) improved the performance of low-light object detection within a specific scene of a low-light mine by combining the dynamic de-fogging enhancement module and image enhancement algorithms with the YOLOv7 network for joint optimization, thus achieving higher detection precision and robustness in low-light and hazy environments.

Although practical, most methods combining low-light image enhancement and object detection models will also inevitably enhance the noise when enhancing the brightness and contrast of low-quality images, affecting the detection effect. Therefore, a denoising algorithm can be introduced to remove image noise before low-light enhancement processing combined with the improved object detection model to extract further and fuse compelling features. Jointly optimizing these steps can achieve higher detection performance and robustness in low-light environments.

## 3 Methodology

### 3.1 Overall network structure

YOLOv7 is an essential version in the YOLO object detection series of models, which has made some optimizations based on the previous versions, introduced more efficient feature extraction and multi-scale feature fusion techniques, and significantly improved the model’s performance. Although the detection precision is slightly lower compared with the subsequent series, such as YOLOv8 (Varghese and M., 2024) and YOLOv9 (Wang et al., 2024a), it shows unique advantages in terms of computational resource requirements, real-time performance, and network structure complexity, etc., and YOLOv7 is still a very competitive model for some resource-constrained applications that require high stability and real-time performance.

YOLOv7x (Wang et al., 2023a) is an enhanced and stabilized version of YOLOv7. YOLOv7x can provide better detection precision and more robust feature representation when dealing with more complex detection tasks. Compared to YOLOv7, YOLOv7x employs a deeper and more complex network structure to improve the feature extraction capability and diversity of detail expression.

**Figure 1:**
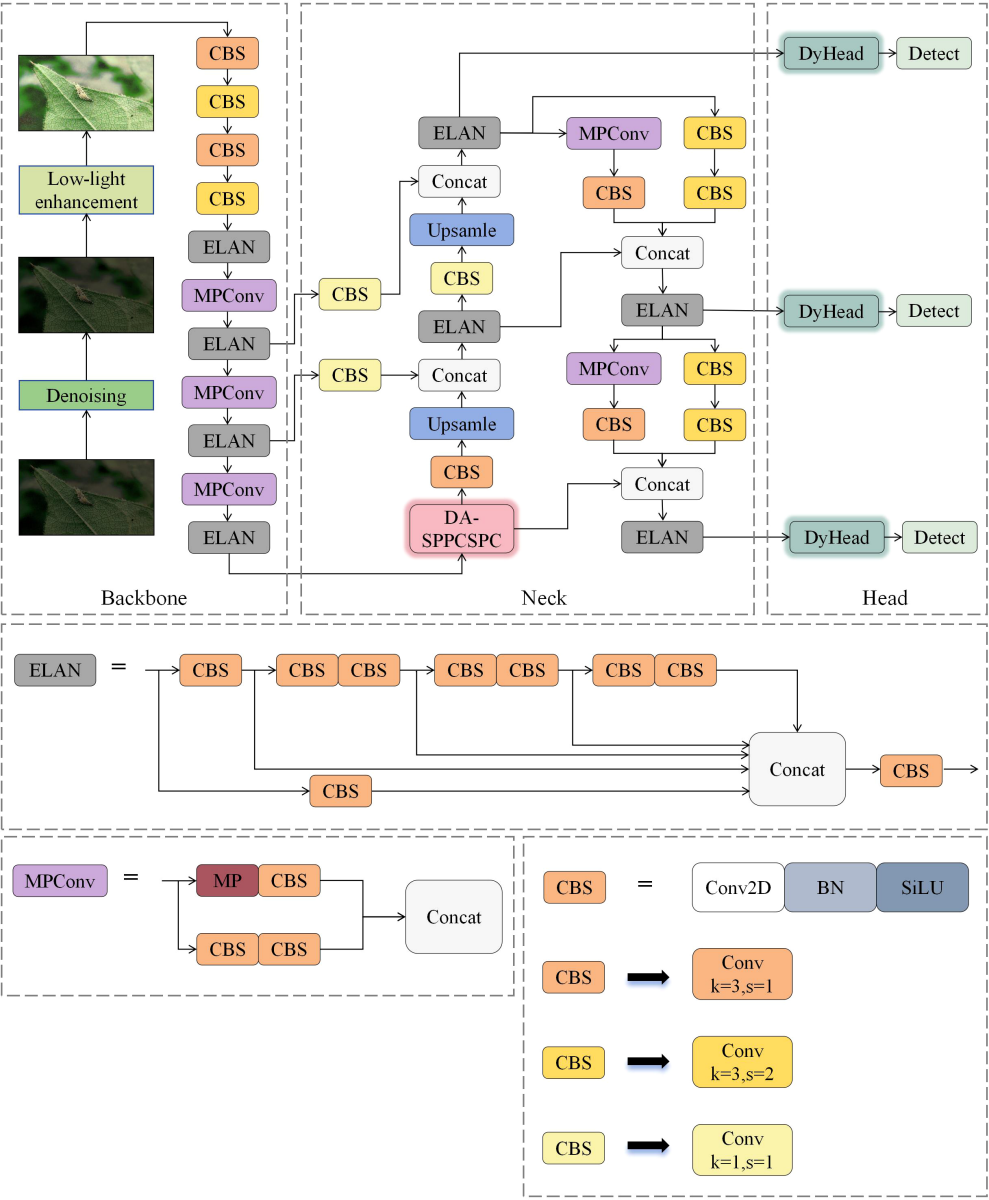
DCP-YOLOv7x overall network structure.

Although YOLOv7x already performs well on most object detection tasks, the performance of YOLOv7x in low-light environments is poor, especially when dealing with data such as insect images, which are small targets. In order to mitigate the effects of low-light environments on the object detection task and optimize the model’s effectiveness in detecting small targets for specific applications, there is a strong need to improve the network architecture of YOLOv7x. The improved network is called DCP-YOLOv7x, and its overall network framework is shown in **Figure 1**.

In the DCP-YOLOv7x network, the denoising and enhancement parts are added. In this paper, FFDNet (Zhang et al., 2017) is used as the denoising algorithm, and EnlighenGAN (Jiang Y. et al., 2021) is used as the low-light enhancement algorithm, which combines the two algorithms to process the low-quality images and to minimize the impact of low-light environments on the object detection task. In the Neck network of the model, the improved DA-SPPCSPC module is used to replace the original SPPCSPC module. Through the introduction of the DAttention mechanism, the weights of the features are dynamically adjusted at different levels and scales so that the network pays more attention to the detailed features in the complex lighting environment and better preserves and enhances the spatial information of the insect to reduce the image distortion and information loss effectively. In addition, the detection head of the model is replaced with DyHead, which is more suitable for this task. DyHead combines dynamic convolution and three attention mechanisms, which can dynamically adjust the structure and parameters of the detection head according to the different sizes, shapes, and numbers of insects, more accurately adjusting the position and size of the detection box and easily distinguishing the color difference between the target and the background, improving the detection precision and generalization ability. Finally, the model’s loss function is optimized by replacing the CIOU loss function originally used in YOLOv7 with the NWD loss function, which has an excellent ability to adapt to scale changes. By introducing normalized weight distances, it pays more attention to the scale differences of target detection boxes and the weight allocation of overlapping areas and performs better for small and dense target detection tasks.

### 3.2 Low quality image enhancement

In this study, we first apply FFDNet for denoising to remove noise from low-quality images, ensuring that extraneous noise, which could negatively impact feature extraction, is minimized. After denoising, we employ EnlightenGAN for low-light image enhancement, improving both the brightness and contrast of the images. The integration of these two techniques is executed in a sequential manner: denoising provides cleaner images, which creates a more stable foundation for the enhancement process to operate effectively. This approach is critical in preventing noise amplification, a common issue when applying enhancement algorithms directly to noisy images.By combining these techniques, we improve overall image quality, which in turn facilitates better performance of the subsequent detection model in low-light environments.

**Figure 2:**
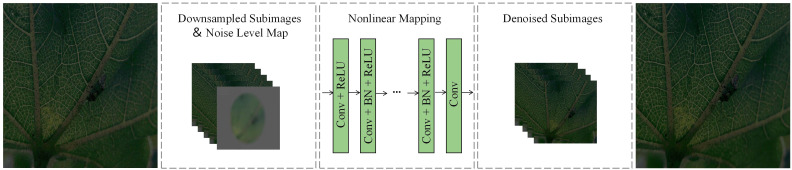
FFDNet network structure (example of Dk-CottonInsect datasets).

In the denoising part, this paper adopts FFDNet as the denoising network.FFDNet is a flexible and funny image-denoising convolutional neural network, which enhances the network’s ability to adapt to various noise situations by introducing a noise level map and combining different noise level information with the input image. The network structure of FFDNet is shown in **Figure 2**, which consists of multiple convolutional and deconvolutional layers, utilizing layer-by-layer feature extraction and reconstruction mechanism to achieve efficient image denoising, and its efficient computational characteristics make it very suitable for real-time image denoising applications.

**Figure 3:**
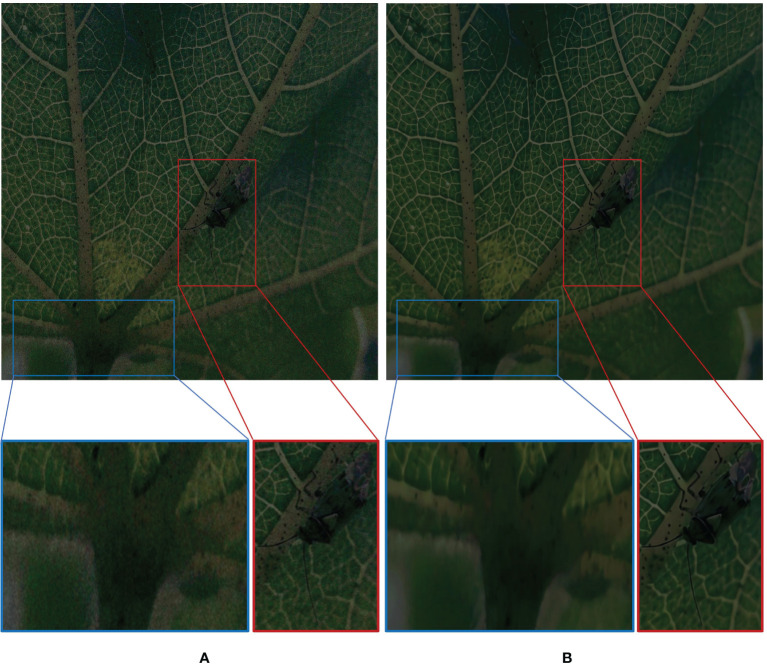
Schematic of denoising effect. **(A)** Before denoising. **(B)** After denoising.

The image-denoising effect of FFDNet is shown in **Figure 3**. By zooming in on the details, it can be seen that the noise of the insect part of the main target of the image is basically removed. The edge information of the insect is presented more clearly, and the contrast between the tiny antennae, the foot, and the downy hairs and the background part is more prominent and more accessible to distinguish.

**Figure 4:**
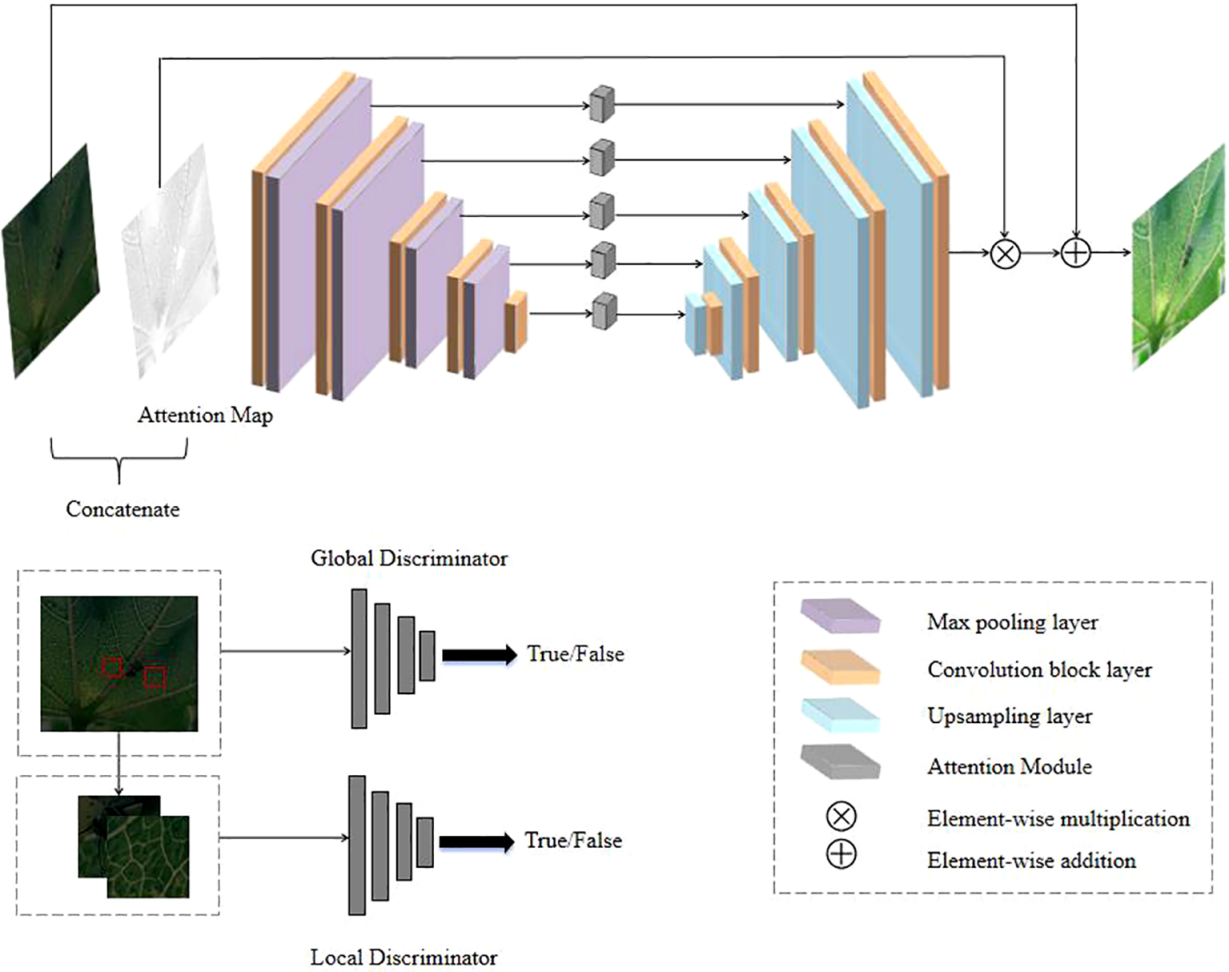
EnlightenGAN network structure (Dk-CottonInsect datasets example).

In the enhancement part, the EnlightenGAN low-light image enhancement algorithm based on GANs(Generative Adversarial Networks) is chosen in this paper. The network structure of EnlightenGAN is shown in **Figure 4**.

Its core architecture includes two parts: a generator and a discriminator. The generator adopts the U-Net structure, which enhances the brightness and contrast of the image through multi-layer convolution and jump connections. The discriminators are divided into two parts: the global discriminator is responsible for evaluating the whole image to ensure that the generated image looks realistic and natural as a whole. The local discriminator randomly crops local areas of the image to ensure that the details and textures of the image can be sharper. In addition, EnlightenGAN introduces a self-supervised learning mechanism that uses a specific loss function to enhance the brightness of the image while maintaining the consistency of the color and details, which can achieve better low-light image enhancement without paired training data and also improve the quality of the image in the case of uneasy to captured normal-light images corresponding to low-light images.

**Figure 5:**
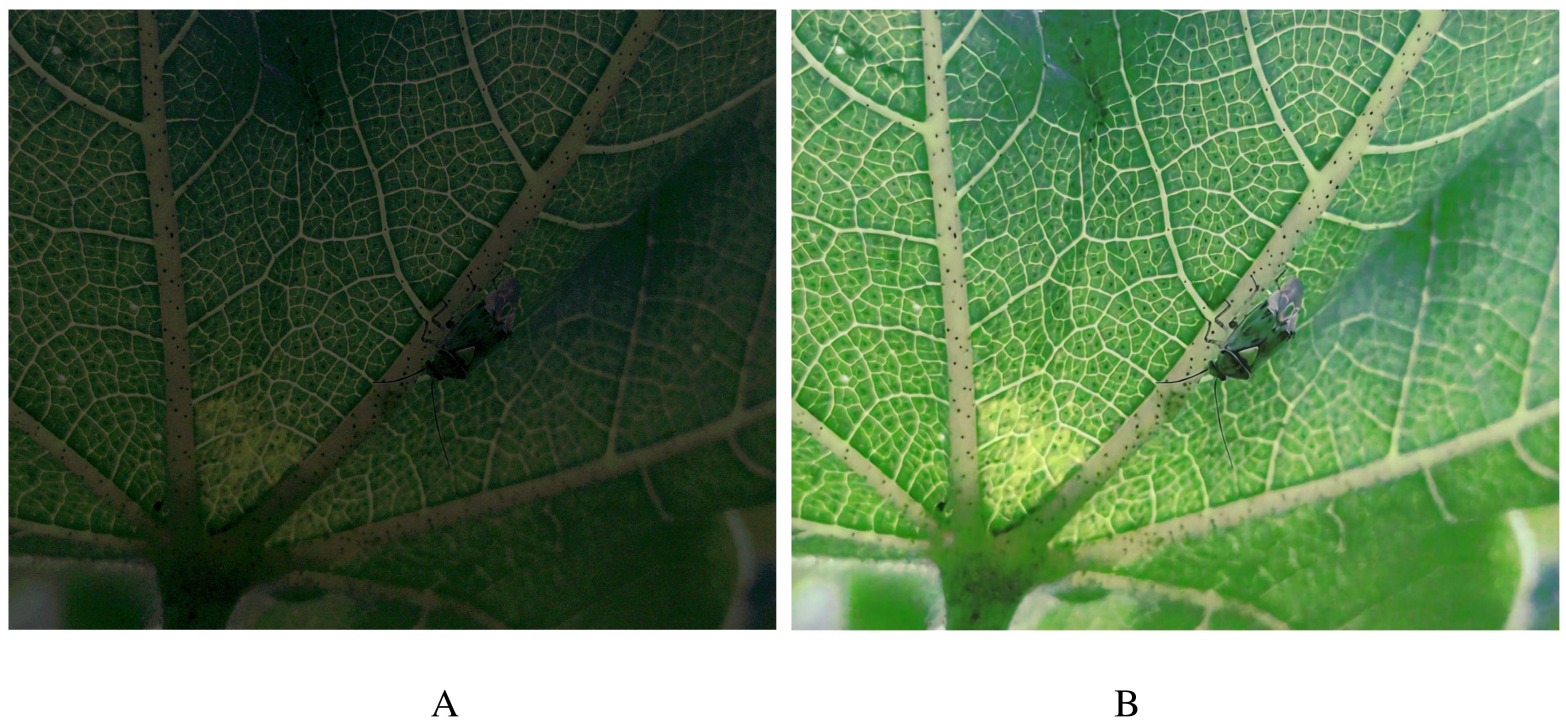
Schematic of low-light enhancement effect. **(A)** Before low-light enhancement. **(B)** After low-light enhancement.

**Figure 5** compares the EnlightenGAN-enhanced low-light image and the original low-quality image. It can be seen that whether it is the image as a whole or each region, the brightness and contrast of the image are balanced, and the colors and details are real and clear, which produces a better visual effect. It is easier to capture the target features, and the difference between the target and background features is more prominent.

### 3.3 DA-SPPCSPC module

The YOLOv7x network uses the SPPCSPC module to enhance multiscale feature extraction and fusion. However, in low-light environments, the image quality is poorer and more noisy, resulting in the traditional CNN being prone to loss of detail information during feature extraction. Although the SPPCSPC module combines multiscale pooling and partial connectivity, the extracted feature information is still insufficient. Moreover, insect’s edge and texture information under low-light environments is more complex, and the key features are weak; the SPPCSPC module may not be able to effectively capture and extract these details when dealing with them, thus affecting the detection precision.

**Figure 6:**
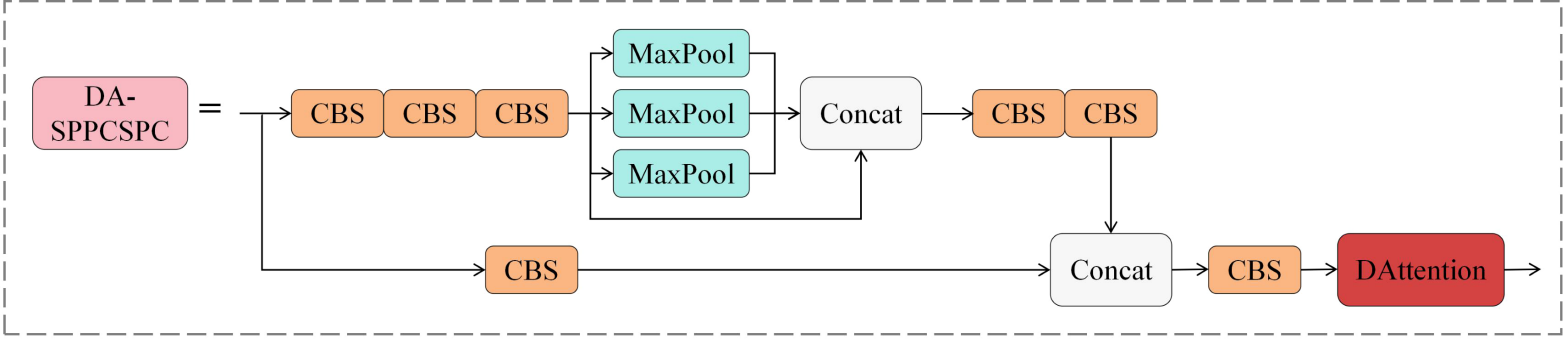
Structure of DA-SPPCSPC module.

In this paper, the DA-SPPCSPC module is proposed to solve the above problems, which separates the features, part of which undergoes convolution operation. The other part undergoes spatial pyramid pooling, which performs multiple maximum pooling operations at different scales on the feature map to capture the information of different receptive fields, then splices the features together and adds the DAttention mechanism in the last layer of the module to improve the feature extraction capability of the model’s ability of the model. The structure of the DA-SPPCSPC module is shown in **Figure 6**.

**Figure 7:**
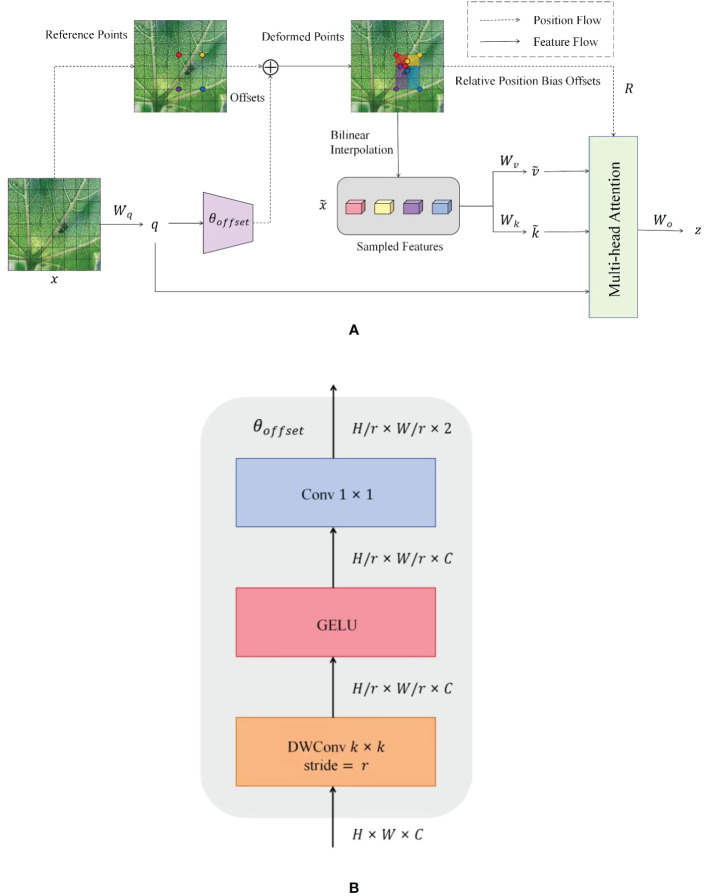
DAttention overall network structure. **(A)** Structure of the DAttention model. **(B)** Offset network structure.

The standard self-attention mechanism processes all the pixels in the image, increasing the computation. DAttention, on the other hand, focuses on only a small number of critical regions in the image. DAttention significantly reduces the amount of computation while maintaining good performance. The DAttention mechanism dynamically selects the sampling points instead of processing the whole image fixedly. This dynamic selection mechanism allows the model to focus more on those regions that are most important to the task at hand and better search for detailed features that are not easily distinguishable. **Figure 7A** illustrates the information flow of DAttention. A set of reference points is first placed on the feature map, and the offsets of these points are learned through an offset generation network. Then, the deformed keys and values are projected from the sampled features based on the deformed points. Relative positional deviations are also computed from the deformed points, enhancing the multi-head attention of the output transformed features. **Figure 7B** shows the detailed structure of the offset generation network. Firstly, the input feature map with size *H* × *W* × *C* is deeply convoluted to obtain the feature map with size *H/r* × *W/r* × *C*. Then, the convoluted feature map is activated nonlinearly by the GELU (Hendrycks and Gimpel, 2016) activation function to enhance the feature expression ability. Then, the number of channels of the feature map is compressed to 2 through the 1 × 1 convolutional layer, and the offset *θ_offset_* of each position of the size *H/r* × *W/r* × 2 is generated, which is used to dynamically adjust the position of the subsequent convolution operation so that the convolution kernel can flexibly adapt to the geometric changes and detailed information in the input feature map.

The model can capture more complex relationships between different parts of the input through this attentional mechanism, thus better performing tasks requiring modeling long-distance dependencies or capturing fine-grained details. DAttention introduces additional learnable parameters learned during the training process through back-propagation, allowing the model to adjust the attentional pattern to better fit the input data. By dynamically adjusting the sampling position, weak features in low-light environments are captured more accurately, improving feature extraction. Adaptive adjustment of weights makes the model pay more attention to local details and improves the ability to capture insect edges and textures.

### 3.4 NWD loss function

YOLOv7x network does not have a detection strategy for tiny targets. Moreover, the YOLOv7x network in the bounding box loss part of the CIOU loss function is used, IOU (Intersection over Union) (Zhou et al., 2019) is mainly used to measure the degree of intersection of the prediction box and the ground truth box, belongs to an index to judge the performance of the model. However, IOU is more sensitive to the positional deviation of low-pixel targets, and a slight positional deviation of low-pixel targets will lead to a significant decrease in IOU, indicating that the target scale of IOU metrics for discrete positional deviations will change, thus giving an impact on the detection task (Li et al., 2024c). Therefore, the CIOU function performs poorly on small object detection. Moreover, there is a problem that it is difficult to converge during training. The size of each image in the training datasets used in this paper is 2000 × 1500 pixels, and the size of some of the insects is below 70 × 70 pixels. Therefore, it is difficult to narrow down the loss value of tiny targets in the bounding box to a specific range using the CIOU loss function, which will lead to a decrease in the performance of the detection model. However, the NWD loss function can better adapt to this scale variation, and the loss value of positional deviation changes more gently. Moreover, NWD can be easily integrated into any anchor-based detection model, which is more capable of detecting small targets than CIOU. Therefore, in this paper, the NWD loss function is used instead of the original CIOU loss function.

The NWD loss function provides a more accurate target localization metric by measuring the normalized Wasserstein distance between the prediction and ground truth box. The Wasserstein distance measures the minimum amount of “work” required to transform one distribution into another. For two distributions P and Q , the Wasserstein distance. W(P,Q) . is defined as:


(1) $$W\left(P,Q\right)={\text{inf}}_{\gamma \;\in \prod^{\;}(P,Q)}E_{\left(x,y\right)∼\gamma }\left[\left\|x-y\right\|\right],$$

Where. $\prod^{\;}(P,Q)$ . is the set of all joint distributions γ with marginal distributions P and Q .

In the object detection task, the prediction box and the ground truth box can be viewed as two distributions. The Wasserstein distance can be used to measure the difference between these two distributions. Assuming that there is a set $\left\{b_{i}^{pred}\right\}$ of prediction boxes and a set $\left\{b_{j}^{gt}\right\}$ of ground truth boxes, the Wasserstein distance between prediction boxes and ground truth boxes can be defined as:


(2) $$W\left(B^{pred},B^{gt}\right)=\frac{1}{N}\sum_{i=1}^{N}{\text{min}}_{j}\|b_{i}^{pred}-b_{j}^{gt}\|,$$

Where $\left\|b_{i}^{pred}-b_{j}^{gt}\right\|$ denotes the distance between the prediction box $b_{i}^{pred}$ and the ground truth box $b_{j}^{gt}$ . The Euclidean Distance metric is usually used.

In order to adapt the Wasserstein distance to the task of object detection at different scales, a normalization process is introduced. The normalized Wasserstein distance, which is also known as NWD loss, can be defined as:


(3) $$NWD\left(B^{pred},B^{gt}\right)=\frac{1}{N}\sum_{i=1}^{N}{\text{min}}_{j}\frac{\left\|b_{i}^{pred}-b_{j}^{gt}\right\|}{size\left(b_{j}^{gt}\right)},$$

Where size $\left(b_{j}^{gt}\right)$ is the size of the ground truth box $b_{j}^{gt}$ , which is used to normalize the distance so that the loss function has a consistent metric for targets of different sizes.

The NWD loss function was chosen for its superior performance in handling small target detection, which is critical in this study. Compared to commonly used loss functions like CIOU and GIOU (Rezatofighi et al., 2019), which focus primarily on the overlap between predicted and ground truth bounding boxes, NWD incorporates the Wasserstein distance, measuring both the position and scale differences between boxes. This makes NWD particularly well-suited for detecting small objects, as it provides a smoother gradient when dealing with slight positional deviations that can significantly affect IOU-based metrics.

Moreover, NWD normalizes the distance based on the size of the ground truth box, which allows it to better capture the relative differences in scale, a key factor when working with small and densely packed targets such as cotton pests. In contrast, traditional IOU-based loss functions are more sensitive to small scale variations, which can lead to slower convergence and less accurate predictions for small objects. This makes NWD a more reliable choice for optimizing object detection models in scenarios where small target accuracy is critical.

By introducing the NWD loss, the original CIOU loss is replaced by NWD loss in the basic loss calculation module and the optimal transmission distribution loss calculation module of YOLOv7x, which improves the detection performance of the model for small and dense targets like insects.

### 3.5 DyHead detection head

Since each predicted position of the detection head of YOLOv7x is independent of the other, the level of feature fusion is limited, and the color of some insects is similar to the background color, it is difficult for the original detection head to distinguish the subtle color differences. Secondly, different insect species have different shapes and sizes, and the locations where the insects stay are randomly distributed. The original detection head has few parameters and limited expression ability, making it challenging to mine the spatial structure information in the features. It lacks an attention mechanism and makes it difficult to dynamically adjust the feature map to highlight targets with different shapes, sizes, and locations, reducing the detection’s precision and reliability. Therefore, in this paper, the detection head of YOLOv7x is improved by introducing the DyHead detection head.

**Figure 8:**
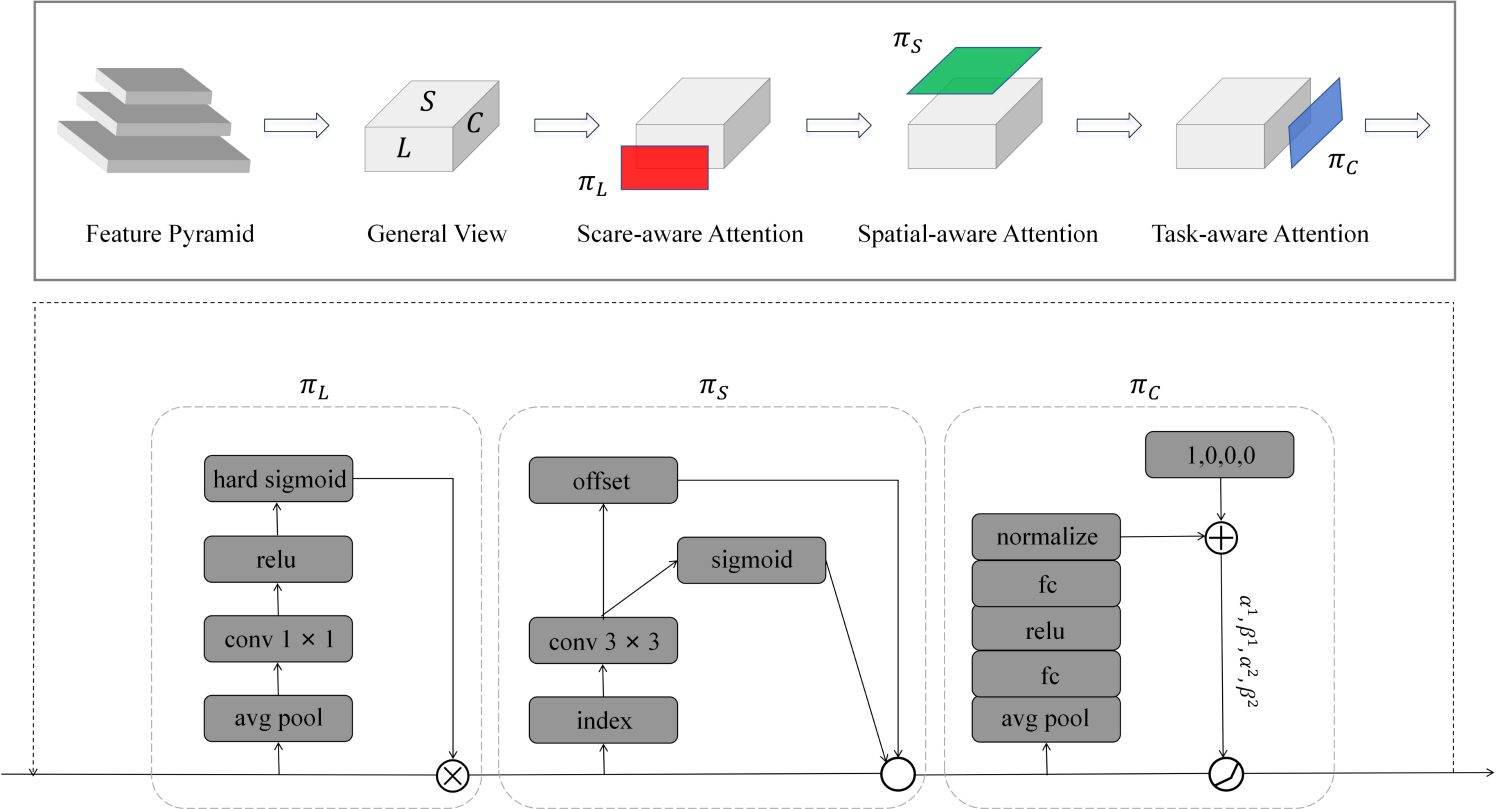
DyHead Module Structure.

The DyHead detection head consists of multiple DyHead modules connected in series. The DyHead module combines scale-aware attention, spatial-aware attention, and task-aware attention and is a dynamic detection head framework whose structure is shown in **Figure 8**.

Specifically, the feature pyramid obtained from the Backbone network needs to be processed into a feature map containing a four-dimensional tensor, which can be denoted as ℱ ∈ ℝ^(^ *^L^* ^×^ *^H^* ^×^ *^W^* ^×^ *^C^* ^)^. *L* represents the number of layers of the feature pyramid, *H* is the height of the feature map, *W* is the width of the feature map, and *C* is the number of channels of the feature map. Defining *S* = *H* × *W*, the feature map contains three dimensions *L*, *S* and *C*, which correspond to scale-awareness, spatial-awareness, and task-awareness, respectively. Its formula can be expressed as:


(4) $$W\left(ℱ\right)=\pi_{C}\left(\pi_{S}\left(\pi_{L}\left(ℱ\right)\cdot ℱ\right)\cdot ℱ\right)\cdot ℱ,$$

(5) $$\pi_{L}\left(ℱ\right)\cdot ℱ=\sigma \left(f\left(\frac{1}{SC}\sum_{S,C}ℱ\right)\right)\cdot ℱ,$$

(6) $$\pi_{S}\left(ℱ\right)\cdot ℱ=\frac{1}{L}\sum_{l=1}^{L}\sum_{k=1}^{K}\omega_{l,k}\cdot ℱ\left(l;p_{k}+\text{Δ}p_{k};C\right)\cdot \text{Δ}m_{k},$$

(7) $$\pi_{C}\left(ℱ\right)\cdot ℱ=\max\;(\alpha^{1}\left(ℱ\right)\cdot {ℱ}_{C}+\beta^{1}\left(ℱ\right),\alpha^{2}\left(ℱ\right)\cdot {ℱ}_{C}+\beta^{2}\left(ℱ\right)),$$

Where ℱ is the input three-dimensional tensor *L* × *S* × *C*. *π_C_* (·)*, π_S_* (·)*, and π_L_* (·) represent the perceptual attention functions on *C*, *S*, and *L*, respectively, applied to ℱ. *σ*(·) is the Hard-Sigmoid function, *f*(·) represents a linear function such as a 1 × 1 convolutional layer, *K* represents the number of sparsely sampled positions, *p_k_* + Δ*p_k_* represents the position where the self-learned spatial offset Δ*p_k_* moves when a discriminant region is selected, ℱ *_C_* is the feature slice of the *C* channel, *α*, *β* are learnable parameters to control the activation threshold.

The DyHead detection head improves the robustness of the model by incorporating multiple attention mechanisms to enhance the model’s detection ability in situations where it is not easy to distinguish the color difference between the target and the background and where the target has diverse morphology and location.

## 4 Experiments and analysis

### 4.1 Datasets setup

This study employs two publicly available datasets to validate the effectiveness of the proposed model. Exdark (Exclusively Dark) (Loh and Chan, 2018) is a standard datasets explicitly designed for object detection in low-light environments. The datasets contains 12 classes of images, including bicycles, buses, cars, cats, dogs, chairs, tables, doors, flowers, vases, people, and road signs, totaling 7363 images. In this paper, 5963 images are used as training sets, 663 images are used as validation sets, and the remaining 737 images are used as test sets. The Exdark datasets is used to verify the effectiveness and robustness of this model in low-light environments.

**Figure 9:**
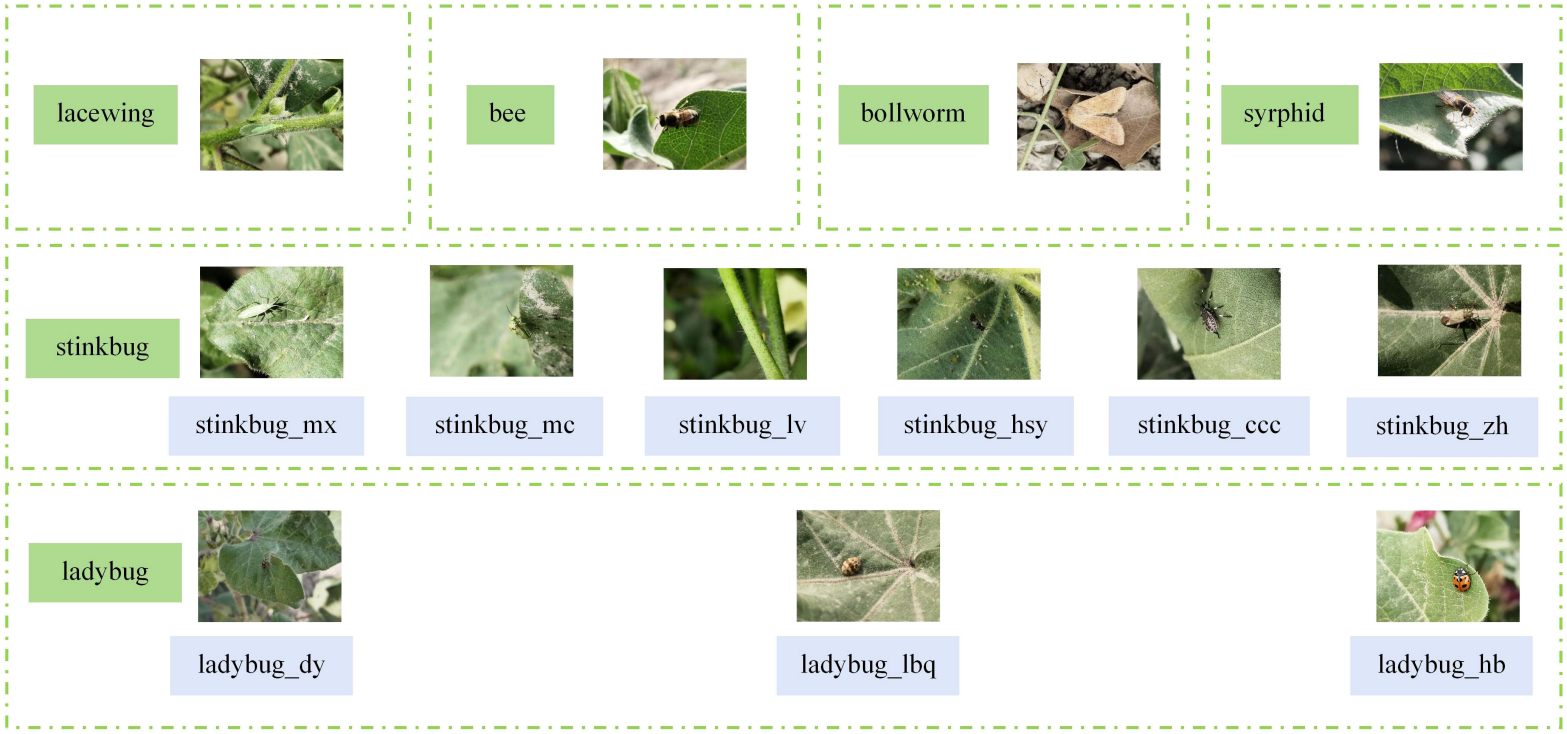
Examples of CottonInsect datasets types of insects.

Another datasets, CottonInsect, is from the China Science Data Center. CottonInsect contains images of major insects in cotton fields in Xinjiang, China, under complex scenarios and is used to identify and detect insects in natural environments. The CottonInsect datasets covers a more comprehensive range of insect species in cotton fields, and the datasets is images collected under normal light. The datasets contains 13 common types of cotton field insects, covering images of different growth periods of insects, totaling 3225 images, and all the insect classifications in this datasets are shown in **Figure 9**.

**Figure 10:**
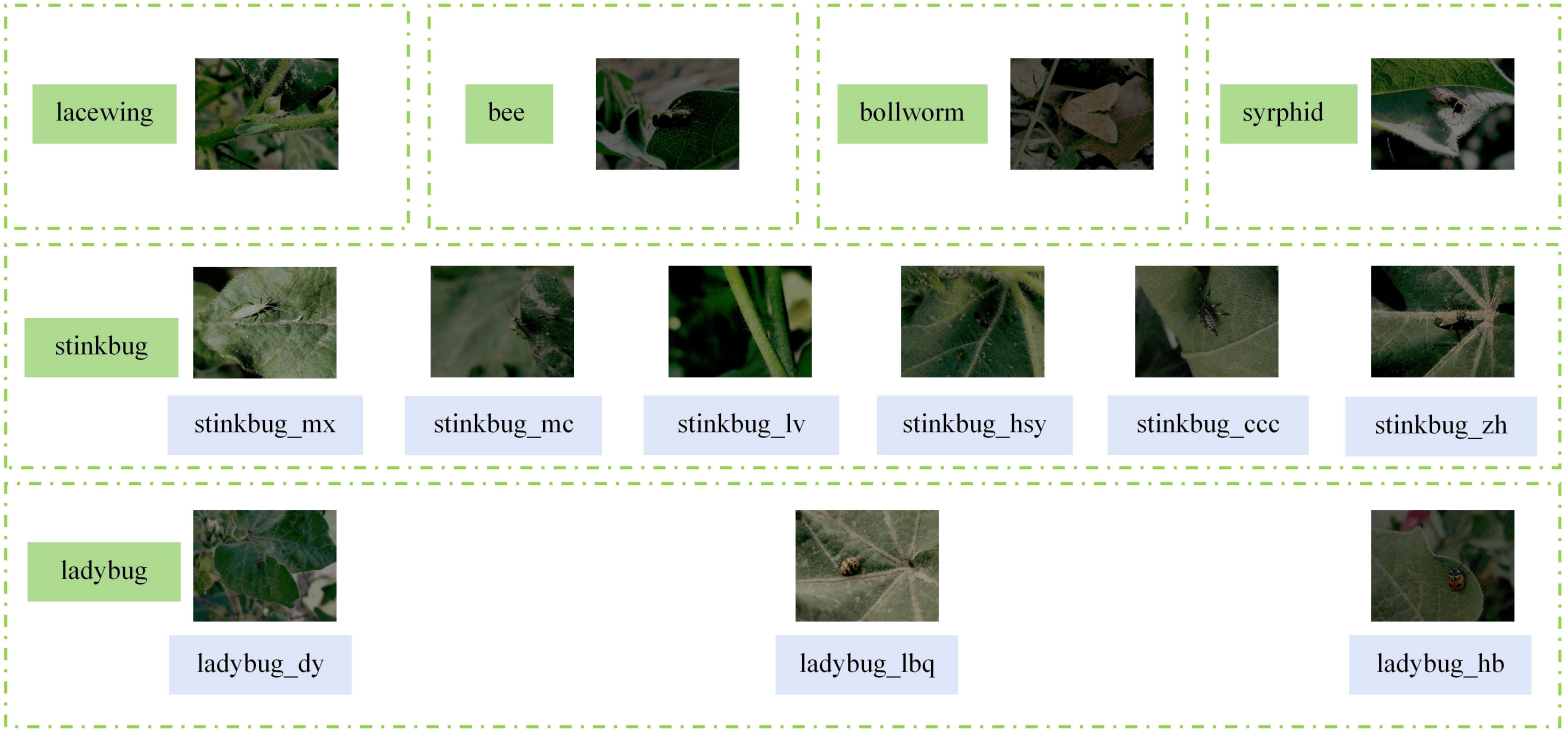
Examples of Dk-CottonInsect datasets types of insects.

Since it is difficult to collect enough insect images in low-light environments, in order to obtain the datasets needed for the experiment, the image degradation method in MAET (Multi-Attribute Enhancement Techniques) (Cui et al., 2022b) is used, Specifically, using the ISP (Image Signal Processor) method, which firstly the image is converted from RGB space to RAW space by reverse ISP, where operations such as randomly reducing the overall brightness and contrast of the image, adding Gaussian noise and spot noise, and adjusting the color balance are performed. Then, the RAW image is converted to a dark RGB image by the forward ISP. This image degradation method can more comprehensively simulate the characteristics of images captured in low-light environments to obtain a more realistic low-quality image datasets, and the effect of the degraded datasets is shown in **Figure 10**. In addition to changing the image quality, the rest of the data format, quantity, and classification have not been changed. The experiment used 2611 images as the training set, 291 as the validation set, and the remaining 323 images as the test set. We refer to the degraded datasets as Dk-CottonInsect.

### 4.2 Evaluation metrics

In order to evaluate the model performance, the experiments used Precision (*P*) (Li et al., 2024d), Recall (*R*) (Li et al., 2024d), Mean Average Precision (mAP) (Li et al., 2024d), and Frames Per Second(FPS) (Sha et al., 2024) as evaluation metrics. The precision is the proportion of classes that are predicted to be positive that are actually positive. The recall is the proportion of the actual positive class the model correctly detects. Mean average precision is an evaluation metric considering *P* and *R* together. In order to better evaluate the detection effect of the model, the mean average precision *mAP*@0.5 when the IOU threshold is 0.5, and the mean average precision *mAP*@0.5: 0.95 when the IOU threshold is averaged between 0.5 and 0.95 are obtained, respectively.

The precision is defined by the formula:


(8) $$P=\frac{TP}{TP+FP},$$

Where *TP* (Li et al., 2024e) denotes the number of samples that were predicted to be in the positive class and were actually also in the positive class. *FP* (Li et al., 2021) denotes the number of samples that were predicted to be in the positive class that were actually in the negative class.

Recall is defined by the formula:


(9) $$R=\frac{TP}{TP+FN},$$

Where *FN* (Li and Liu, 2023) denotes the number of samples in which the positive class was incorrectly predicted to be negative.

The mean average precision is defined by the formula:


(10) $$AP=\int_{0}^{1}P\left(R\right)dR,$$

(11) $$mAP=\frac{1}{n}\sum_{i=1}^{n}AP_{i},$$

Where *P*(*R*) (Li et al., 2022a) denotes the precision at different recall rates and n denotes the number of classes.

Frames per second is defined by the formula:


(12) $$FPS=\frac{1000}{time},$$

Where *time* denotes the time required by the model to process a single image,measured in milliseconds.

### 4.3 Experimental environment and setup

This experiment uses an AMD Ryzen 7 5800X processor and a single RTX 3060 graphics card with 12 GB for model training. Linux system, CUDA 12.2, Python 3.8, and Pytorch 2.1.0 are used for software configuration. The parameters of the training phase are set as follows: the image size is, 640 × 640, the SGD optimizer is used, the initial learning rate is 0.01, the momentum factor is 0.937, the weight decay is 0.0005, and 200 rounds of training are conducted with batch size 8.

In this study, the setting of hyperparameters has undergone thorough experimental validation and optimization. The learning rate is set to 0.01, utilizing a gradient warming and step decay strategy to ensure the model’s rapid convergence and stable performance while avoiding issues such as gradient explosion and vanishing gradients. When dealing with high-noise data in complex low-light environments, a smaller batch size allows for more effective updates of model parameters, enhancing the adaptability to data distributions and generalization ability. By combining existing research on the optimization of deep learning models with observations of model performance during the experiments, the batch size is ultimately set to 8, balancing computational efficiency and model accuracy, especially in accurately detecting objects in challenging low-light conditions. The momentum factor is set to 0.937, and the weight decay coefficient is set to 0.0005. The selection of these parameters is derived from grid search optimization, aiming to enhance the model’s generalization ability and effectively prevent overfitting. The choice of these hyperparameters ensures the robustness and efficiency of the model in complex environments.

### 4.4 Different image enhancement methods performance comparison

To evaluate the effectiveness of the adopted image enhancement methods, different image enhancement techniques were applied to the Dk-CottonInsect and Exdark datasets, and corresponding indicators were assessed. For the Dk-CottonInsect datasets with reference images, Peak Signal-to-Noise Ratio (PSNR) (Zhang et al., 2020), Structural Similarity Index (SSIM) (Zhang et al., 2020), Learned Perceptual Image Patch Similarity (LPIPS) (Zhang et al., 2020) and Naturalness Image Quality Evaluator(NIQE) (Demir and Kaplan, 2023) metrics were used to comprehensively quantify the quality of enhanced images. **Table 1** presents the specific performances of the Dk-CottonInsect datasets under these quantitative evaluation metrics.

**Table 1:** Image quality evaluation after enhancement of the Dk-CottonInsect datasets.

Methods	PSNR↑	SSIM↑	LPIPS↓	NIQE↓
ZeroDCE(2020)	18.25	0.65	0.52	0.0614
SCI(2022)	18.24	0.60	0.62	0.0667
FFDNet+EnlightenGAN(Ours)	18.63	0.69	0.43	0.0528

"↑" indicates that a higher indicator value corresponds to better image quality, while "↓" indicates that a lower indicator value corresponds to better image quality.

The experimental results indicate that, although ZeroDCE (Guo et al., 2020) and SCI (Ma et al., 2022) perform well on certain low-light image quality metrics, their performance on all indicators is inferior to the FFDNet+EnlightenGAN image enhancement method we employed. Particularly in terms of detail preservation and image naturalness, our enhancement method demonstrates superior performance, providing better support for subsequent object detection tasks.

On the Exdark datasets, which lacks reference images, the Naturalness Image Quality Evaluator (NIQE) (Demir and Kaplan, 2023), Perception-based Image Quality Evaluator (PIQE) (Choudhary et al., 2023), and Blind/Referenceless Image Spatial Quality Evaluator (BRISQUE) (Choudhary et al., 2023) were used to evaluate the quality of enhanced images. These no-reference image quality evaluators provided a comprehensive assessment, reflecting various aspects such as naturalness, perceptual quality, and spatial distortions in the enhanced images. **Table 2** presents the specific performances of the Exdark datasets under these quantitative evaluation metrics.

**Table 2:** Image quality evaluation after enhancement of the Exdark datasets.

Methods	NIQE↓	PIQE↓	BRISQUE↓
ZeroDCE(2020)	0.048	15.248	20.572
SCI(2022)	0.037	18.327	24.387
FFDNet+EnlightenGAN(Ours)	0.029	11.620	17.343

"↑" indicates that a higher indicator value corresponds to better image quality, while "↓" indicates that a lower indicator value corresponds to better image quality.

From **Table 2**, the FFDNet + EnlightenGAN image enhancement method demonstrated the best performance on the Exdark datasets compared to the other two methods, achieving the highest scores across all three quality metrics. This result indicates that FFDNet+EnlightenGAN effectively enhances the naturalness, perceptual quality, and spatial coherence of the images, which are critical in low-light conditions.

Overall, the FFDNet+EnlightenGAN image enhancement method we employed offers a more significant advantage in overall quality improvement and detection performance support.

### 4.5 Experimental comparison of different attention mechanisms

To select the most suitable attention mechanism for this task, we systematically evaluated the impact of several different attention mechanisms on model performance using the Dk-CottonInsect datasets, including Attlepe (Attentive Pooling attention) (Santos et al., 2016),DAttention (Xia et al., 2022),Biformer attention (Zhu et al., 2023), and CPCA (Channel Prior Convolutional Attention) (Huang et al., 2024). From **Table 3**, it can be observed that certain attention mechanisms resulted in a decline in detection performance due to increased complexity or inappropriate focus, indicating that not all attention mechanisms are suitable for object detection in low-light environments. In contrast, DAttention consistently demonstrated significant performance improvements across various experimental settings, showcasing its exceptional adaptability.

**Table 3:** Comparison of different attention mechanisms with YOLOv7x on Dk-CottonInsect datasets.

Methods	*P*(%)	*R*(%)	*mAP*@0.5(%)	*mAP*@0.5: 0.95(%)
YOLOv7x	81.5	75.5	79.8	68.0
YOLOv7x+Attlepe(2016)	80.5	74.6	79.9	56.3
**YOLOv7x+DAttention(2022)**	**94.0**	**85.9**	**91.5**	**69.4**
YOLOv7x+Biformer(2023)	94.4	79.3	91.3	65.3
YOLOv7x+CPCA(2024)	93.7	71.1	84.4	57.1

The bold values represent the highest performance for each indicator, as well as the attention mechanism we ultimately selected.

This enhancement can be attributed to the ability of DAttention to dynamically adjust attention weights, allowing the model to effectively prioritize critical features, such as edges and textures, that are often obscured under low-light conditions. This selective enhancement of features greatly improves the detection accuracy of objects in complex environments. Therefore, DAttention was chosen as the optimal attention mechanism for our low-light object detection model, significantly addressing the challenges posed by degraded image quality and laying a solid foundation for achieving higher detection performance.

### 4.6 Comparison experiment on the number of DyHead modules

Different numbers of DyHead modules can be set in the DyHead detection head, and different numbers of modules will affect the performance of the whole model. In order to pick the most suitable number of DyHead modules to balance the performance and computational cost of the model, DyHead module comparison experiments were conducted. The experiments were conducted on the Dk-CottonInsect datasets when the number of DyHead modules was 1, 2, 4, and 6, respectively, and the results of the experiments are shown in **Table 4**.

**Table 4:** Performance comparison of different number of DyHead modules on Dk-CottonInsect datasets.

Amount	P(%)	R(%)	mAP@0.5(%)	mAP@0.5:0.95(%)	*FPS*
1	85.2	75.7	79.2	55.5	109.8
2	90.2	83.5	89.8	71.6	79.1
4	89.9	81.3	87.2	73.8	50.4
6	87.3	78.6	85.0	68.7	18.1

According to the experimental results, it can be found that the number of DyHead modules is not the more the better. The model performance is better when the number of DyHeads is 2 and 4. By comparison, although *mAP*@0.5: 0.95-value is highest when 4 DyHeads, *P*-value, *R*-value, and *mAP*@0.5-value are higher when the number of DyHeads is 2 than when it is 4. Moreover, **Table 4** shows that as the number of DyHead modules increases, *FPS* decreases significantly. When using 2 DyHeads, the model achieves 79.1 *FPS*, which ensures both detection accuracy and real-time processing capabilities. However, with 4 DyHeads, the *FPS* drops to 50.4, and further to 18.1 with 6 DyHeads. Since *FPS* represents the number of frames processed per second, a higher *FPS* is generally better.Therefore, considering the computational cost and the real-time detection demand of the task, the number of DyHeads is finally chosen as 2 in this experiment.

### 4.7 Performance analysis of the training process

**Figure 11:**
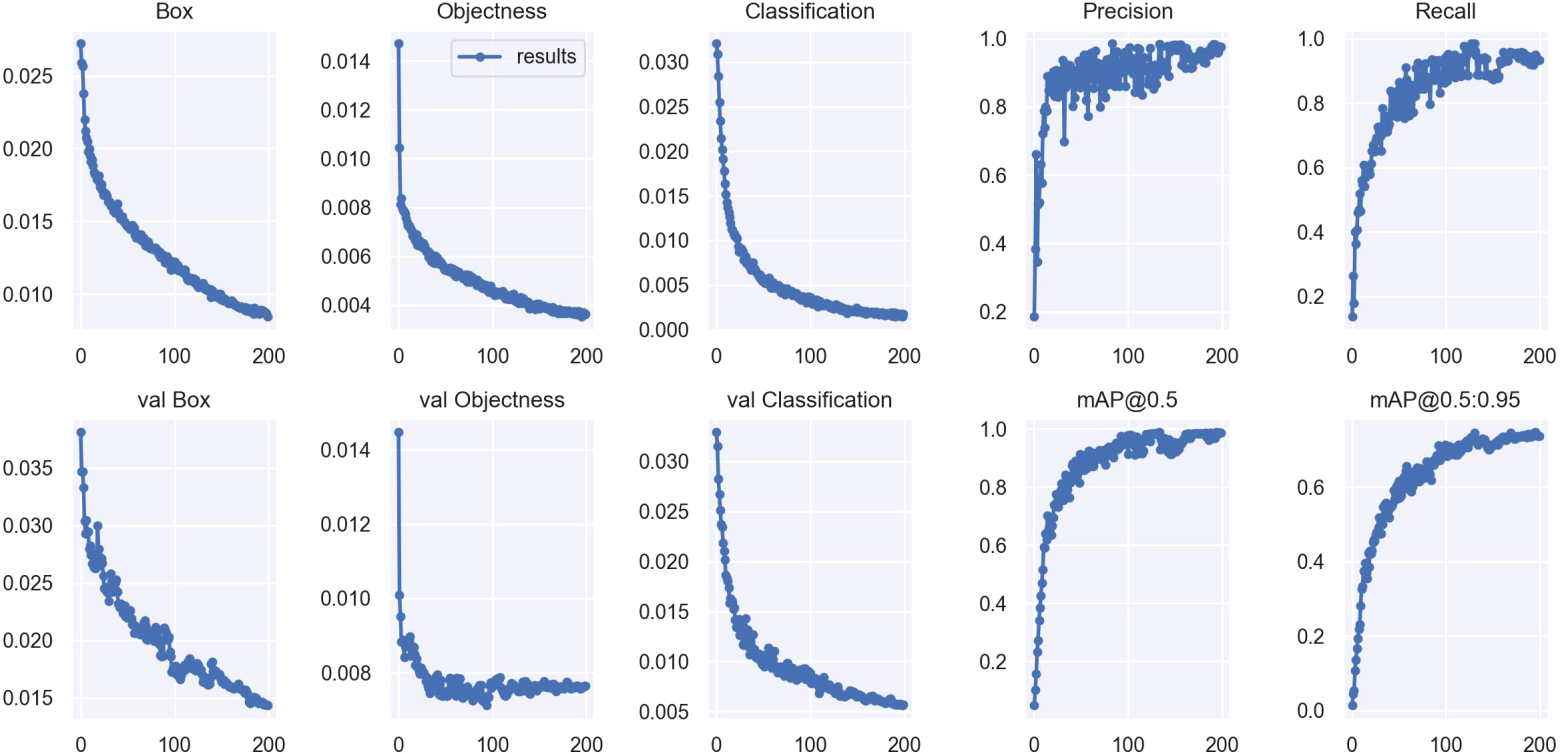
DCP-YOLOv7x performance metrics changes.

In this study, the DCP-YOLOv7x model was trained on the Dk-CottonInsect datasets for 200 epochs, and the metrics changes on the training and validation sets during the training process are shown in **Figure 11**.

The first three columns in **Figure 11** are the bounding box regression loss, Objectness prediction loss, and Classification loss, respectively. The first row is the change in the loss function during training, and the second is the change during validation, with the x-axis denoting the epochs and the y-axis denoting the loss values. From **Figure 11**, it can be seen that with the increase of epochs, the loss (Box, Objectness, and Classification) of training and validation decreases gradually and levels off, and it can be seen that the model is not overfitted, which indicates that the fitting performance and stability of this model is strong. The last two columns represent the changes in the precision, recall, and mean average precision of the model, respectively, with the x-axis representing epochs and the y-axis representing the values of each index. The figure shows that the precision and recall rate gradually increase with the increase of epochs and tend to be stable, indicating that the model’s performance in accurately identifying and detecting targets constantly improves and finally reaches a stable state. The mean average precision also continues to improve with the increase of epochs, i.e., the model’s overall detection performance is excellent under different IOU thresholds, and it has a strong generalization ability. Overall, this model is effective and stable in the training process.

**Figure 12:**
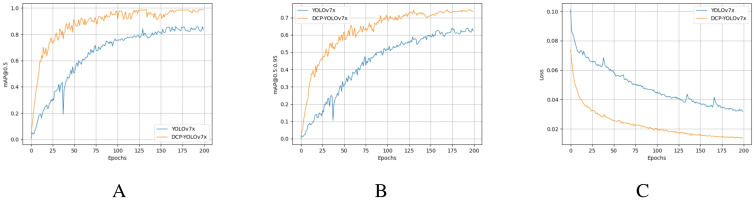
Training mAP and total loss. **(A)** Training mAP@0.5-values. **(B)** Training mAP@0.5:0.95-values. **(C)** Training total loss-values.

**Figure 12** shows the trend of DCP-YOLOv7x and YOLOv7x (Wang et al., 2023a) training mAP-values and total loss-values on the Dk-CottonInsectt datasets. As seen from **Figures 12A, B**, the mAP-value of DCP-YOLOv7x continued to increase during the training process and remained stable at about 170 epochs. In addition, the mAP-value of DCP-YOLOv7x is always higher than that of YOLOv7x during the training process, which proves the effectiveness of the proposed method. As seen from (**Figure 12C**), the loss-values of both models are trending downward and have converged around 200 for epochs. DCP-YOLOv7x converges faster and has a lower loss-value than YOLOv7x. By comparing the mAP-value and the total loss-value, it can be seen that the proposed DCP-YOLOv7x model has good learning and optimization ability, and the detection performance of the model has also reached a high level.

### 4.8 Discussion and comparison of experimental results on Exdark datasets

In order to verify that the DCP-YOLOv7x model proposed in this paper has better detection performance in different low-light environments, we compared DCP-YOLOv7x with IAT-YOLO (Cui et al., 2022a), DLN-YOLO (Lim et al., 2022), DE-YOLO (Qin et al., 2022),YOLOv7x (Wang et al., 2023a), DK YOLOv5 (Wang et al., 2023b), NLE-YOLO (Peng et al., 2024) and various versions of the current mainstream YOLOv7. We conducted experiments on the Exdark datasets, and the Dk-CottonInsectt datasets, respectively, and the detection results of various object detection models on the Exdark datasets are shown in **Table 5**.

**Table 5:** Performance comparison of different models on Exdark datasets.

Methods	*P*(%)	*R*(%)	*mAP*@0.5(%)	*mAP*@0.5: 0.95(%)
IAT-YOLO(2022)	76.3	61.7	68.4	39.8
DLN-YOLO(2022)	77.9	62.1	69.8	41.2
DE-YOLO(2022)	70.3	43.9	51.0	25.6
YOLOv7x(2023)	71.7	62.0	69.7	41.5
DK_YOLOv5(2023)	78.2	64.5	71.3	43.4
NLE-YOLO(2024)	74.7	62.9	71.9	46.7
DCP-YOLOv7x(Ours)	82.1	74.0	80.5	52.7

As can be seen from **Table 5**, the DCP-YOLOv7x model proposed in this paper shows the best results in all the four metrics of *P*, *R*, *mAP*@0.5, and *mAP*@0.5: 0.95, which illustrates the superiority of the model. Among the other four algorithms, DK YOLOv5 has the highest *P* and *R*- values of 78.2% and 64.5%, respectively, and NLE-YOLO has the highest *mAP*@0.5 and *mAP*@0.5: 0.95 values of 71.9% and 46.7%, respectively. The DCP-YOLOv7x model in this paper has a P-value of 82.1%, an *R*-value of 74.0%, a *mAP*@0.5-value of 80.5%, and a *mAP*@0.5: 0.95-value of 52.7%, which are 3.9% and 9.5% higher than that of DK YOLOv5, and 8.6% and 6.0% higher than that of NLE-YOLO, respectively. Compared with the baseline, the indexes were improved by 10.4%, 12%, 10.8%, and 11.2%, respectively. The comparison results with other methods show that the present model has a strong feature extraction ability in different low-light environments and has excellent detection performance.

**Figure 13:**
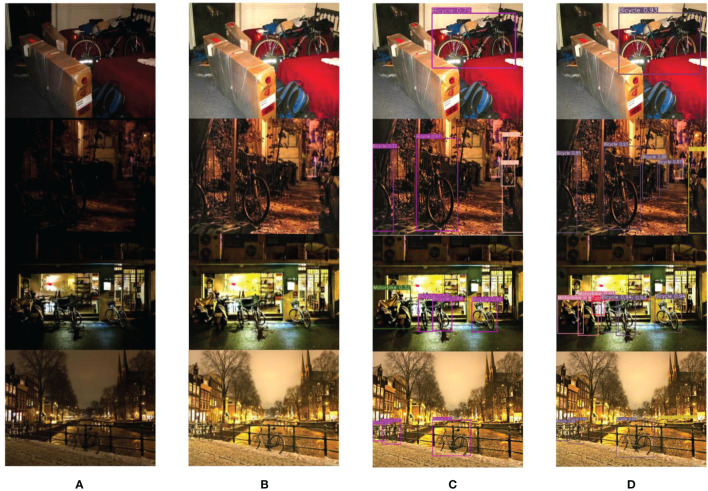
Comparison of detection results on the Exdark datasets (Loh and Chan, 2018). **(A)** Original low-quality image. **(B)** Enhanced image. **(C)** YOLOv7x detection results. **(D)** DCP-YOLOv7x detection results.

**Figure 13** are visual comparison charts of the detection results from different models on the Exdark datasets, respectively. In **Figure 13**, (A) is the original low-quality image, (B) is the image after denoising and low-light enhancement, (C) is the detection effect of YOLOv7, and (D) is the detection effect of the DCP-YOLOv7x model.

**Figure 13** demonstrates the superiority of DCP-YOLOv7x in different low-light environments and different tasks. As can be seen from the figure, the YOLOv7x has a relatively high missed detection rate, and there are many small targets near or far away that are not detected.

### 4.9 Discussion and comparison of experimental results on Dk-CottonInsect datasets

In order to verify that the present model is indeed effective and reliable when facing the task of insect object detection in low-light environments, we conducted comparison experiments with YOLOv7 (Wang et al., 2023a),YOLOv7-tiny (Wang et al., 2023a), and YOLOv7x (Wang et al., 2023a) on the Dk-CottonInsect datasets, and the experimental results are shown in **Table 6**.

**Table 6:** Performance comparison of different models on Dk-CottonInsect datasets.

Methods	*P*(%)	*R*(%)	*mAP*@0.5(%)	*mAP*@0.5: 0.95(%)
YOLOv7(2023)	72.2	72.4	80.1	62.0
YOLOv7-tiny(2023)	81.2	72.6	79.4	59.7
YOLOv7x(2023)	81.5	75.5	79.8	68.0
DCP-YOLOv7x(Ours)	95.9	91.2	95.4	71.5

As can be seen from **Table 6**, the DCP-YOLOv7x model in this paper exhibits the highest detection performance compared to other YOLO models. The *P*-value, *R*-value, *mAP*@0.5-value, and *mAP*@0.5: 0.95-value of the DCP-YOLOv7x model are 95.9%, 91.2%, 95.4%, and 71.5%, respectively. The *P*-value, *R*-value, and *mAP*@0.5: 0.95-value are improved by 14.4%, 15.7%, and 3.5% compared to the secondhighest YOLOv7x, and the *mAP*@0.5-value is improved by 15.3% over the second-highest YOLOv7. So the present model can still extract the fine features of small and dense targets well even when facing poor lighting conditions, minimizing the interference of low-quality images on the detection task.

**Figure 14:**
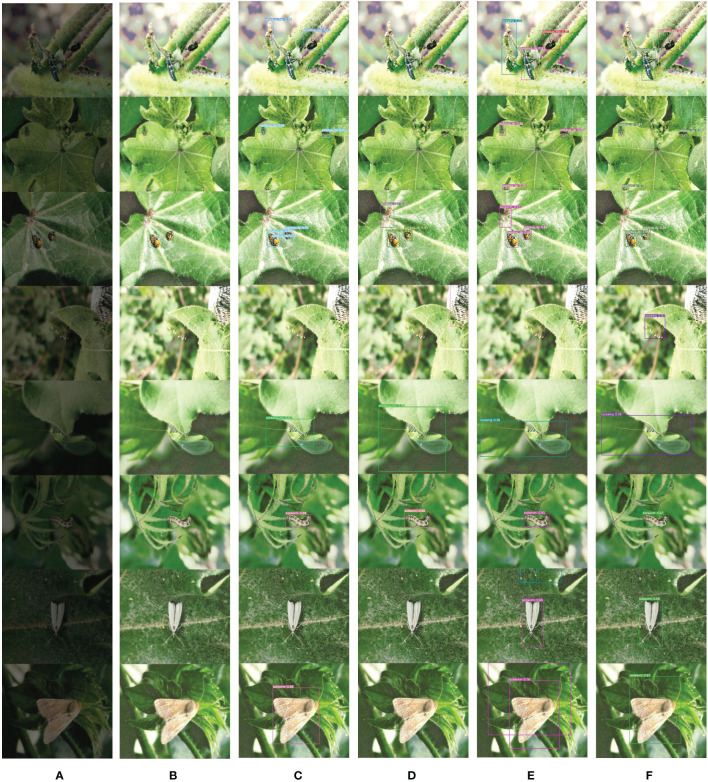
Comparison of detection results on the Dk-CottonInsect datasets. **(A)** Original low-quality image. **(B)** Enhanced image. **(C)** YOLOv7 detection results. **(D)** YOLOv7-tiny detection results. **(E)** YOLOv7x detection results. **(F)** DCP-YOLOv7x detection results.

**Figure 14** are visual comparison charts of the detection results from different models on the DkCottonInsect datasets, respectively. In **Figure 14**, (A) is the original low-quality image, (B) is the image after denoising and low-light enhancement, (C) shows the detection effect of YOLOv7, (D) shows the detection effect of YOLOv7-tiny, (E) shows the detection effect of YOLOv7x, and (F) shows the detection effect of DCP-YOLOv7x.

As can be seen from **Figure 14**, the DCP-YOLOv7x model proposed in this paper performs better in practical applications than the other three models. In the first three rows of the image, there are multiple targets of the same or different classes, and some of them overlap each other. YOLOv7x and YOLOv7-tiny predict two overlapping targets into one target, while DCP-YOLOv7x not only correctly detects all classes and targets, but also distinguishes overlapping targets, and the confidence level of prediction is higher than that of YOLOv7. The fourth, fifth, and last three rows are several forms of the two insects at different stages of growth, respectively. As can be seen from the figure, none of the other three models detected one of the lacewing morphologies, YOLOv7 and YOLOv7-tiny did not detect one or two morphologies of bollworm, while DCP-YOLOv7x detected all morphologies of both insects with high confidence. And the other three models have a relatively high false detection rate. In the first, third, fourth, seventh, and eighth lines of the image, the other three models incorrectly detected a part of the background as a target. In addition, YOLOv7-tiny also has a large degree of missed detection, not detecting all or part of the target in many images.

Through intuitive comparison, the DCP-YOLOv7x model effectively improves the accuracy of multiscale object detection tasks in different low-light environments, and the model has good generalization performance and can be applied to a variety of tasks.

### 4.10 Discussion and comparison of experimental results on CottonInsect datasets

To assess whether the model can detect insects with the same high accuracy and efficiency under normal exposure, we also conducted comparisons on the CottonInsect datasets with various versions of the mainstream YOLOv7 model. The specific experimental results are shown in **Table 7**.

**Table 7:** Performance comparison of different models on CottonInsect datasets.

Methods	*P*(%)	*R*(%)	*mAP*@0.5(%)	*mAP*@0.5: 0.95(%)
YOLOv7(2023)	90.4	80.5	89.5	69.6
YOLOv7-tiny(2023)	88.0	89.8	89.3	71.8
YOLOv7x(2023)	85.1	87.0	89.9	71.5
DCP-YOLOv7x(Ours)	95.6	95.1	96.5	80.2

From **Table 7**, it can be seen that, compared to other models, the DCP-YOLOv7x model in this paper demonstrates the highest detection performance. The *P*-value, *R*-value, *mAP*@0.5-value, and *mAP*@0.5: 0.95-value of the DCP-YOLOv7x model are 95.6%, 95.1%, 96.5%, and 80.2% respectively. The *R*-value and *mAP*@0.5: 0.95-value are 5.3% and 8.4% higher than those of the second-highest YOLOv7-tiny, the *P*-value is 5.2% higher than that of YOLOv7, and the *mAP*@0.5-value is 6.6% higher than that of YOLOv7x. This indicates that under normal exposure conditions, the DCP-YOLOv7x model, with its optimized feature extraction network and detection structure, can more accurately capture insect features, reducing both false positives and missed detections.

**Figure 15:**
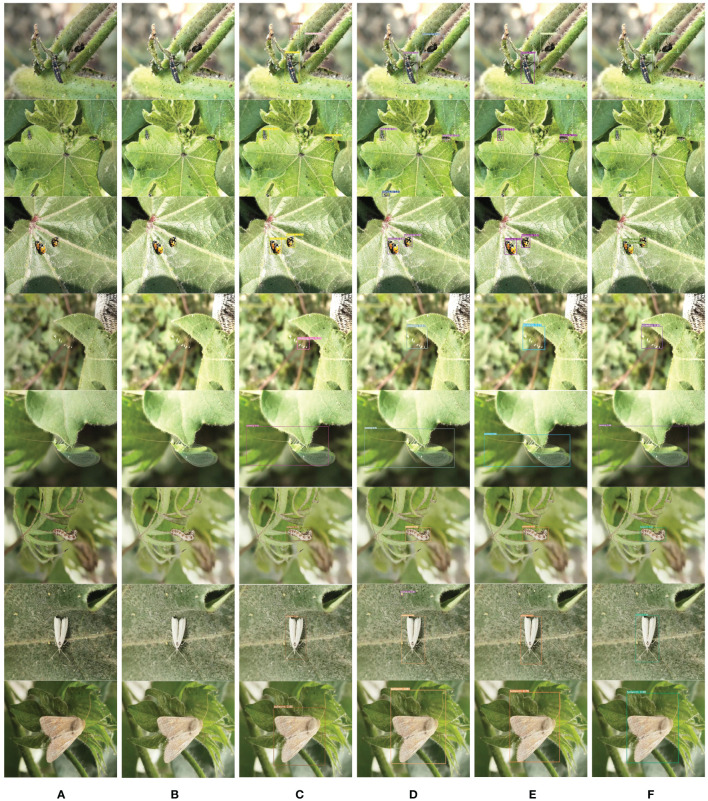
Comparison of detection results on the CottonInsect datasets. **(A)** Original low-quality image. **(B)** Enhanced image. **(C)** YOLOv7 detection results. **(D)** YOLOv7-tiny detection results. **(E)** YOLOv7x detection results. **(F)** DCP-YOLOv7x detection results.

**Figure 15** are visual comparison charts of the detection results from different models on the CottonInsect datasets, respectively. In **Figure 15**, (A) is the original low-quality image, (B) is the image after denoising and low-light enhancement, (C) shows the detection effect of YOLOv7, (D) shows the detection effect of YOLOv7-tiny, (E) shows the detection effect of YOLOv7x, and (F) shows the detection effect of DCP-YOLOv7x.

**Figure 15** illustrates that under normal exposure conditions, DCP-YOLOv7x still achieves excellent detection performance. While YOLOv7, YOLOv7-tiny, and YOLOv7x can detect the approximate location and type of the target, they struggle with overlapping objects and do not accurately define target boundaries. Detection boxes fail to completely encompass all insect parts, such as antennae and wings, and confidence scores are relatively low. In contrast, DCP-YOLOv7x captures all parts of the insects while maintaining a high confidence level, demonstrating its effectiveness and superiority in detecting insects under normal exposure conditions.

### 4.11 Ablation experiments

We verify the effect of the improved module on the model performance through ablation experiments, and the results of the ablation experiments for each module on the ExDark and Dk-CottonInsect datasets are shown in **Tables 8**, **9**, respectively. In **Tables 8**, **9**, *A* denotes the denoising and low-light image enhancement algorithms, *B* denotes the DA-SPPCSPC module, *C* denotes the NWD loss, and *D* denotes the DyHead detection head.

**Table 8:** Ablation results for Exdark datasets.

Methods	*P*(%)	*R*(%)	*mAP*@0.5(%)	*mAP*@0.5: 0.95(%)
YOLOv7x	71.7	62.0	69.7	41.5
YOLOv7x+A	74.9	67.3	73.8	44.2
YOLOv7x+A+B	76.4	69.4	75.1	46.1
YOLOv7x+A+B+C	78.1	72.5	78.1	49.8
YOLOv7x+A+B+C+D (DCP-YOLOv7x)	82.1	74.0	80.5	52.7

**Table 9:** Ablation results for the Dk-CottonInsect datasets.

Methods	*P*(%)	*R*(%)	*mAP*@0.5(%)	*mAP*@0.5: 0.95(%)
YOLOv7x	81.5	75.5	79.8	68.0
YOLOv7x+A	93.9	78.1	91.3	62.7
YOLOv7x+A+B	94.0	85.9	91.5	69.4
YOLOv7x+A+B+C	95.2	91.2	93.1	70.4
YOLOv7x+A+B+C+D (DCP-YOLOv7x)	95.9	91.2	95.4	71.5

From the results in **Table 8**, it can be seen that when all the modules are added, DCP-YOLOv7x performs the most excellent, which shows that all the improvements carried out in this paper are essential. Compared to the baseline, the *P*-value, *R*-value, *mAP*@0.5-value, and *mAP*@0.5: 0.95-value increased by 3.2%, 5.3%, 4.1%, and 2.7%, respectively, after combining the denoising and low-light image enhancement algorithms, which proves that combining the denoising and low-light enhancement algorithms can reduce the impact of the luminance, contrast, and noise problems generated by low-light environments on the task. After adding the DA-SPPCSPC module, the four values increased by 1.5%, 2.1%, 1.3%, and 1.9%, respectively, compared with the previous ones, indicating that the DA-SPPCSPC module can effectively enhance the feature extraction ability of the model in complex environments to obtain better detection performance. After introducing the NWD loss to optimize the loss function of the object detection network, the four values increased by 1.7%, 3.1%, 3.0%, and 3.7%, respectively, which verifies the multi-scale dynamic feature fusion ability of the NWD loss, shows better performance when there are multiple targets of different scales in the image, and improves the generalization of the model. After the model detection head was finally replaced with DyHead, the performance indexes of this paper’s model DCP-YOLOv7x reached the highest, four values are 82.1%, 74.0%, 80.5%, and 52.7%, which are 4%, 1.5%, 2.4%, and 2.9% higher than the previous ones, respectively, indicating that the three attention mechanisms introduced by the DyHead are of great help to the situation of diverse target shapes and variable positions, and the detection performance of the model is improved by fusing different attention mechanisms.

The results in **Table 9** further illustrate the effectiveness of the improvement modules in dealing with the low-light pest detection task. After adding all the improvement modules, the various indexes of this model reach the optimum, and the *P*-value, *R*-value, *mAP*@0.5-value, and *mAP*@0.5: 0.95-value are 95.9%, 91.2%, 95.4%, and 71.5%, which verifies that the DCP-YOLOv7x model proposed in this paper can sufficiently cope with the situation of insufficient light on the insect detection task. Each improvement module has a relatively positive impact on the model.

## 5 Conclusion

In this paper, an object detection model DCP-YOLOv7x for detecting cotton pests in low-light environments is proposed to cope with the problems of degradation of image quality, features not easy to extract, and low detection precision caused by low-light environments. The DCP-YOLOv7x model uses the FFDNet method for denoising the image to eliminate background noise and the EnlightenGAN low-light image enhancement algorithm to enhance the brightness and contrast of the image. The enhanced image is used as an input to the improved object detection network to improve the image quality while enhancing the feature extraction capability of the model. We replace the SPPCSPC module in the model Neck network with the DA-SPPCSPC module that carries the DAttention mechanism, which enhances the model’s ability to extract the edge and texture features that are not easily accessible. In addition, NWD is introduced to optimize the loss function of the model and enhance the model’s detection performance for small and dense targets. Finally, the detection head of the model is modified to a DyHead detection head that mixes three attention mechanisms to improve the model’s ability to cope with targets with diverse morphology and variable locations. In addition, since low-light pest datasets are challenging to collect, this paper adopts an image degradation method to acquire the Dk-CottonInsect low-light cotton pest datasets. The experimental results proved that the DCP-YOLOv7x model proposed in this paper has higher detection precision in different low-light environments and can better fulfill the task of cotton pest detection in low-light environments, which is of practical application value.

The DCP-YOLOv7x model demonstrates considerable performance improvements in detecting cotton pests under low-light conditions; however, challenges remain in highly complex environments, such as cases with significant occlusions that can reduce detection accuracy. Additionally, the integration of denoising and image enhancement techniques in the modified detection network increases the model’s computational demands, presenting deployment challenges in real-time, resource-constrained settings like edge devices, drones, or low-power agricultural hardware.In practical deployment for large-scale agricultural scenarios, it is also necessary to consider enhancing the model’s scalability across different crop types, environmental conditions, and pest species.To address these issues, future work will focus on further optimizing the computational efficiency of the model to facilitate its deployment in real-time, resource-constrained environments, such as agricultural edge devices. Additionally, expanding the DkCottonInsect datasets with more diverse lighting conditions, pest species, and field scenarios will be essential to improving the model’s robustness and generalization capability. These efforts aim to ensure that the DCP-YOLOv7x model can maintain high detection performance across a wider range of practical agricultural applications.

## Data Availability

Publicly available datasets were analyzed in this study. This data can be found here: ExDark Database: https://github.com/cs-chan/Exclusively-Dark-Image-Dataset CottonInsect Database: https://www.scidb.cn/en/detail?dataSetId=3f36bce8e41849a6a33e34fb0f8ae581.
